# Supplementary material for: Homozygous receptors for insulin and not IGF-1 accelerate intimal hyperplasia in insulin resistance and diabetes
Source: Nat Commun. 2019 Sep 27;10:4427. doi: 10.1038/s41467-019-12368-2 (PMC6765023; doi:10.1038/s41467-019-12368-2)
Supplement: Supplementary file 1 — Supplementary Information [file 41467_2019_12368_MOESM1_ESM.pdf]

Supplementary Table 1. RNA-sequencing results of WT or SMIGF1RKO VSMCs stimulated with insulin

SMIGF1RKO insulin vs WT insulin

	SMIGF1RKO	WT		
	Ins vs Con FC	Ins vs Con FC	DeltaDelta FC	DeltaDelta p
Egln3	4.25	1.69	2.51	0.0109
S1pr3	3.3	1.33	2.48	0.0813
Slc16a3	4.04	1.74	2.32	0.000532
Rcor2	2.76	1.38	2	0.0257
Adamts14	2.41	1.31	1.83	0.00119
Tubb3	2.85	1.58	1.81	0.00177
Pfkl	2.53	1.44	1.76	0.000197
Pfkfb3	2.86	1.66	1.73	0.0131
Relt	2.97	1.8	1.65	0.00949
Gys1	2.3	1.4	1.64	0.002
Has2	2.23	1.37	1.63	0.146
Kif26b	2.4	1.48	1.62	0.0142
Trabd2b	2.65	1.69	1.57	0.021

Supplementary Figure 1

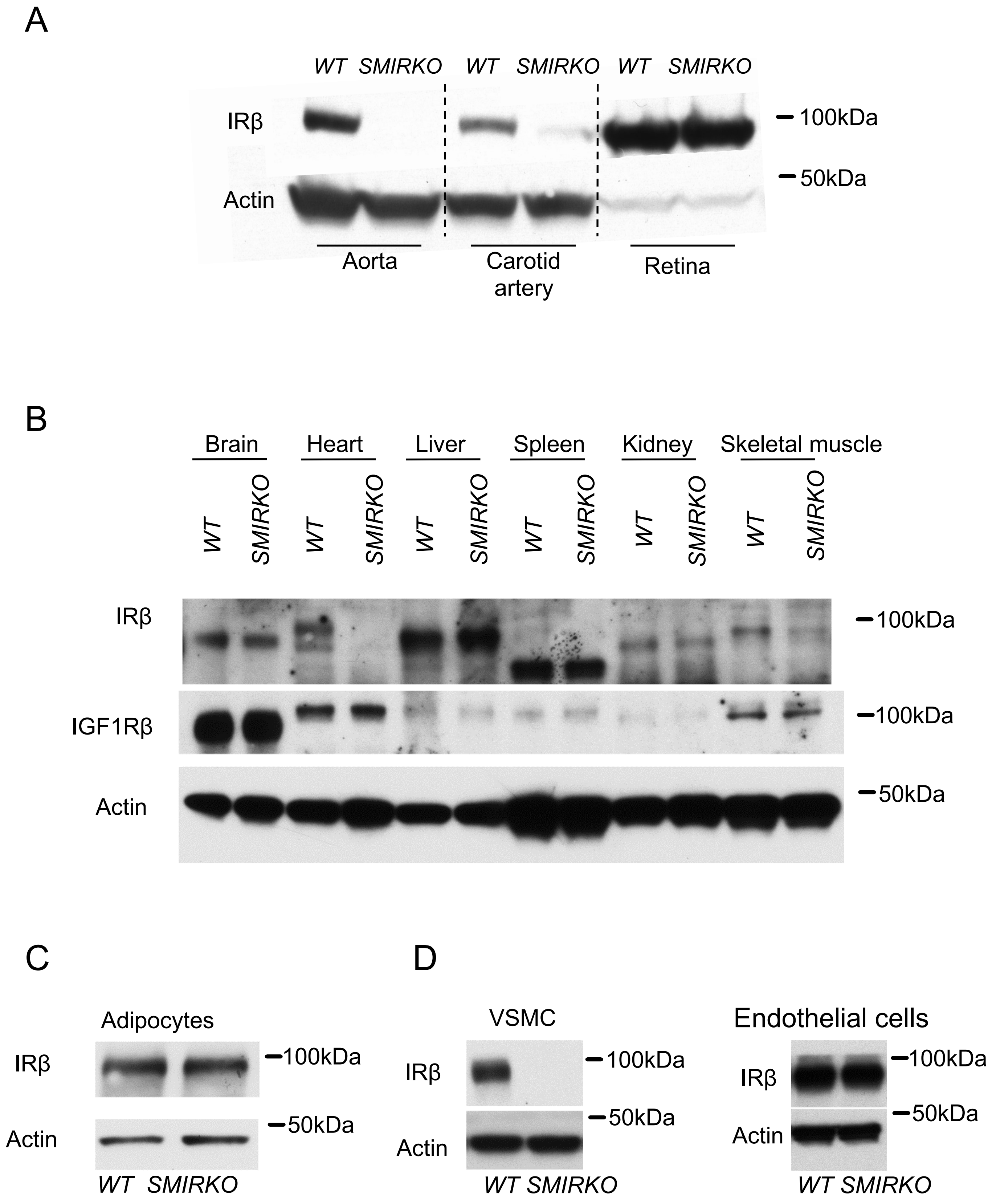

Supplementary Figure 2

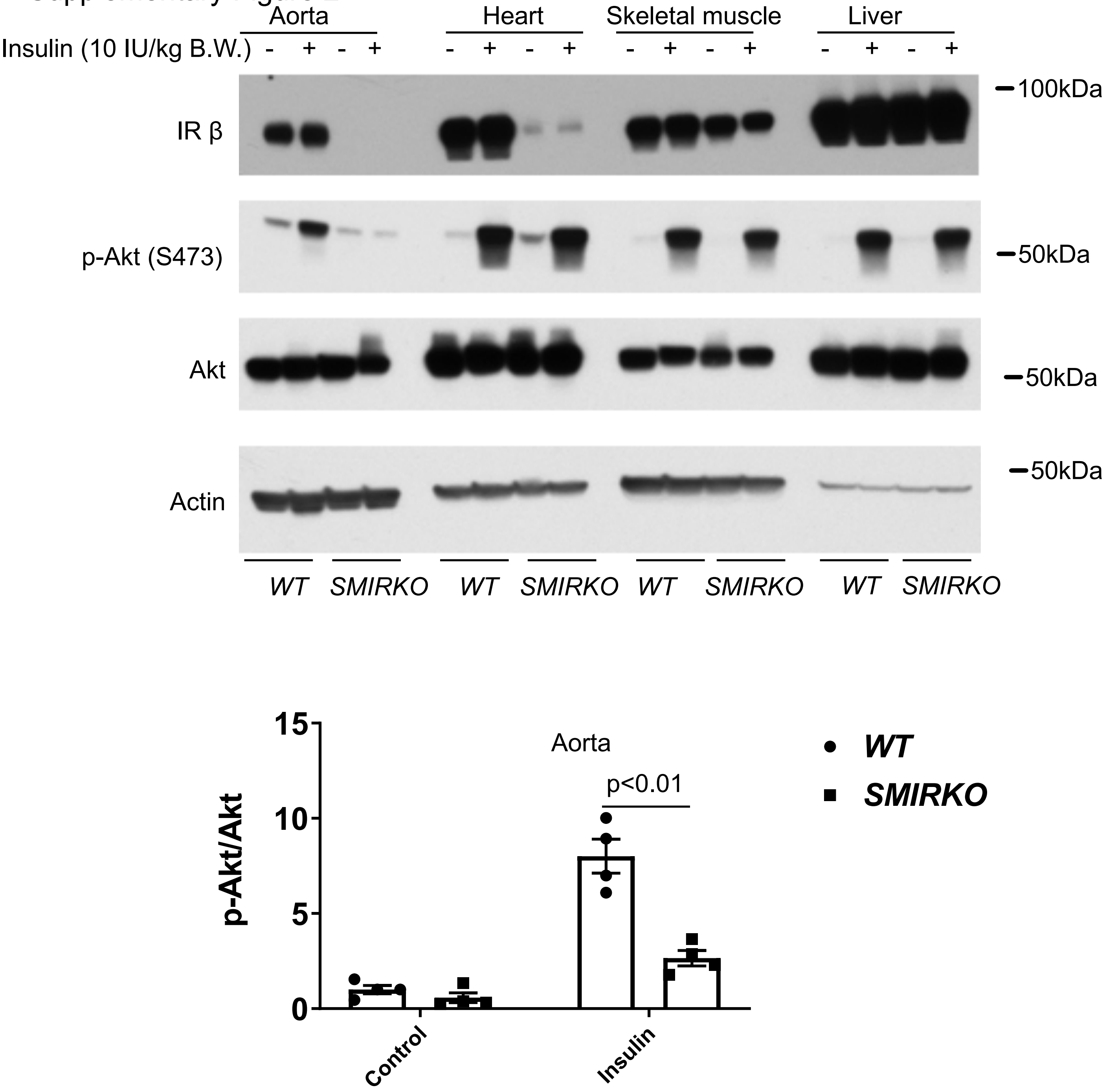

Supplementary Figure 3

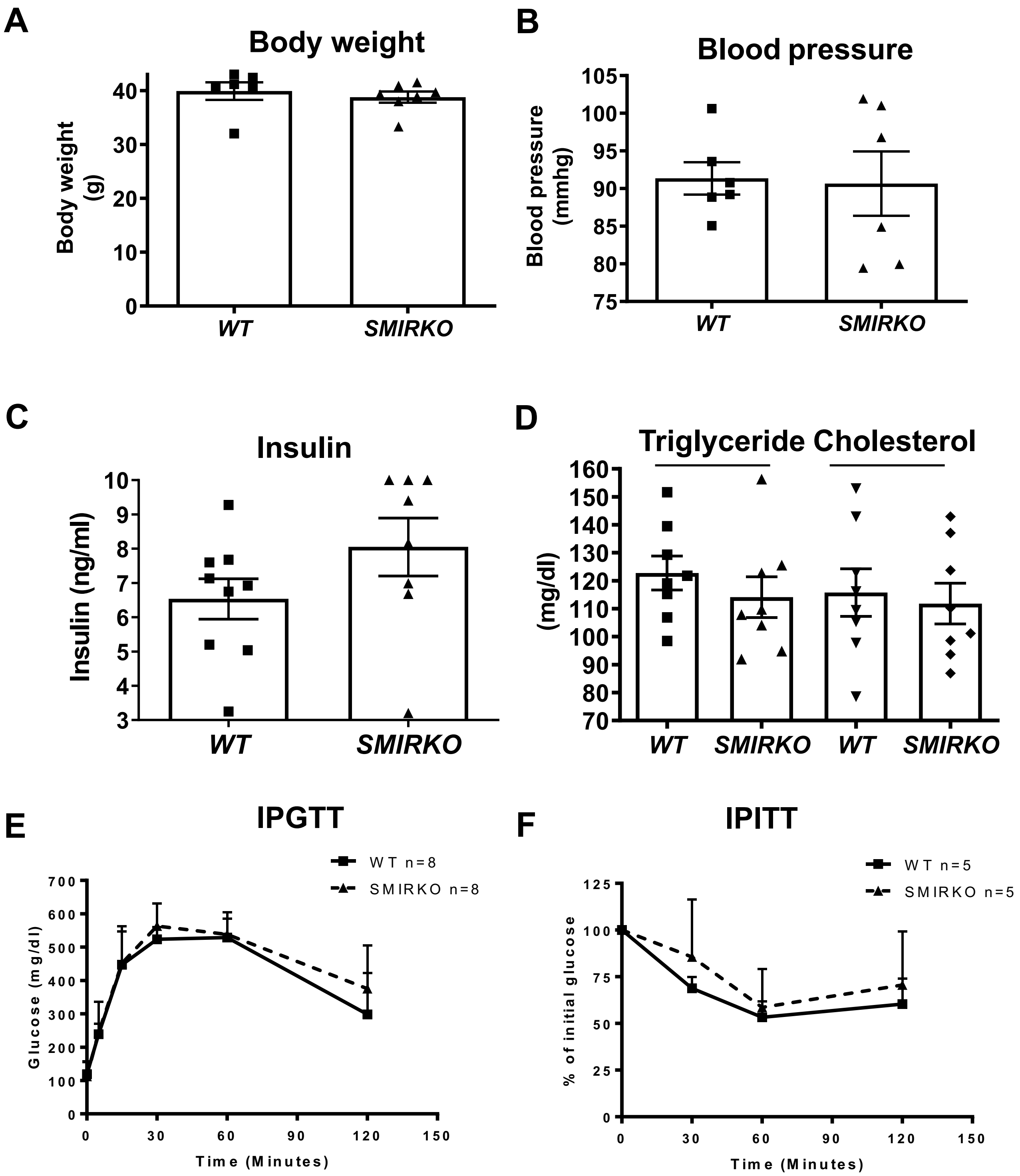

Supplementary Figure 4

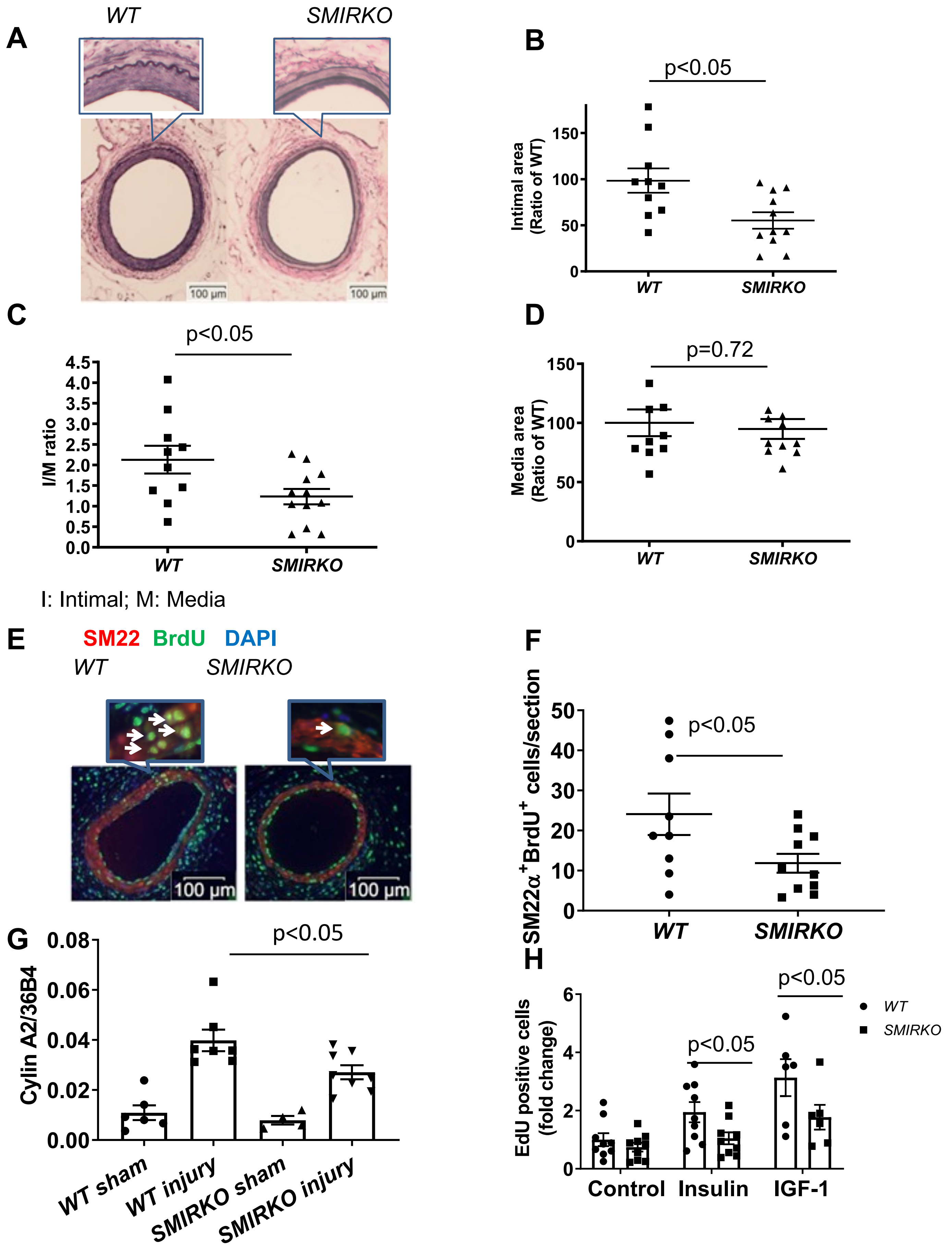

Supplementary Figure 5

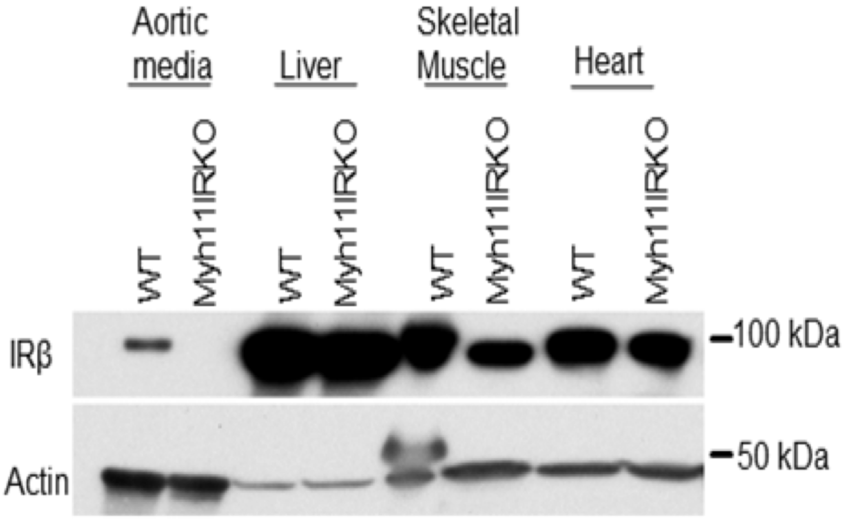

Supplementary Figure 6

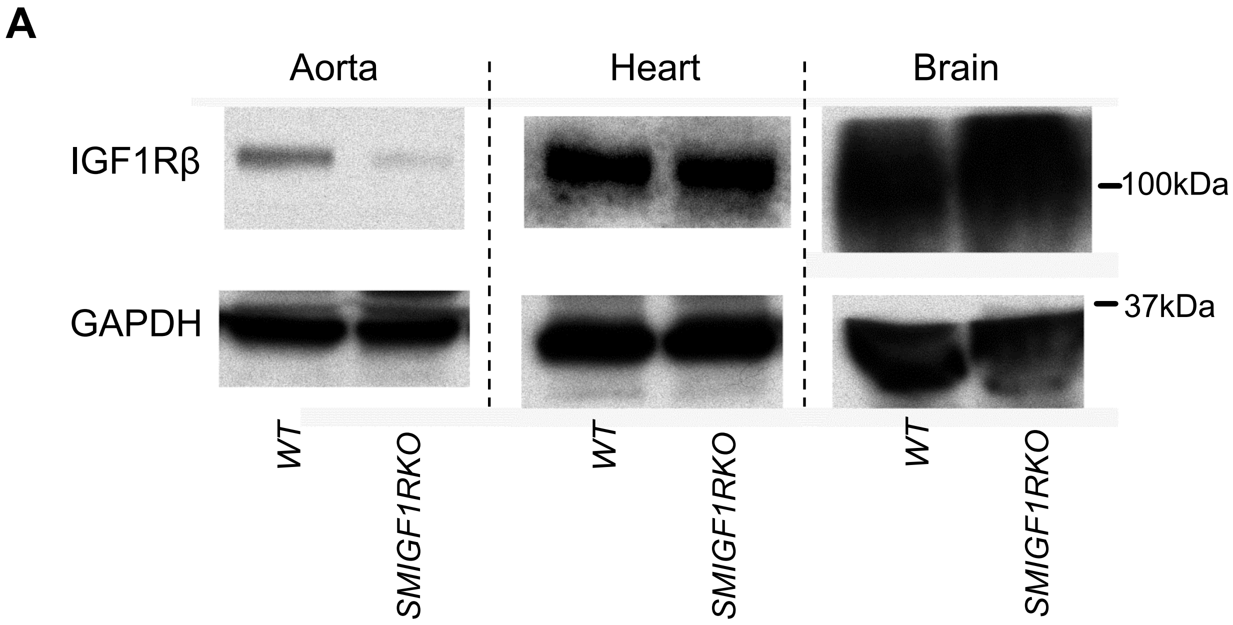

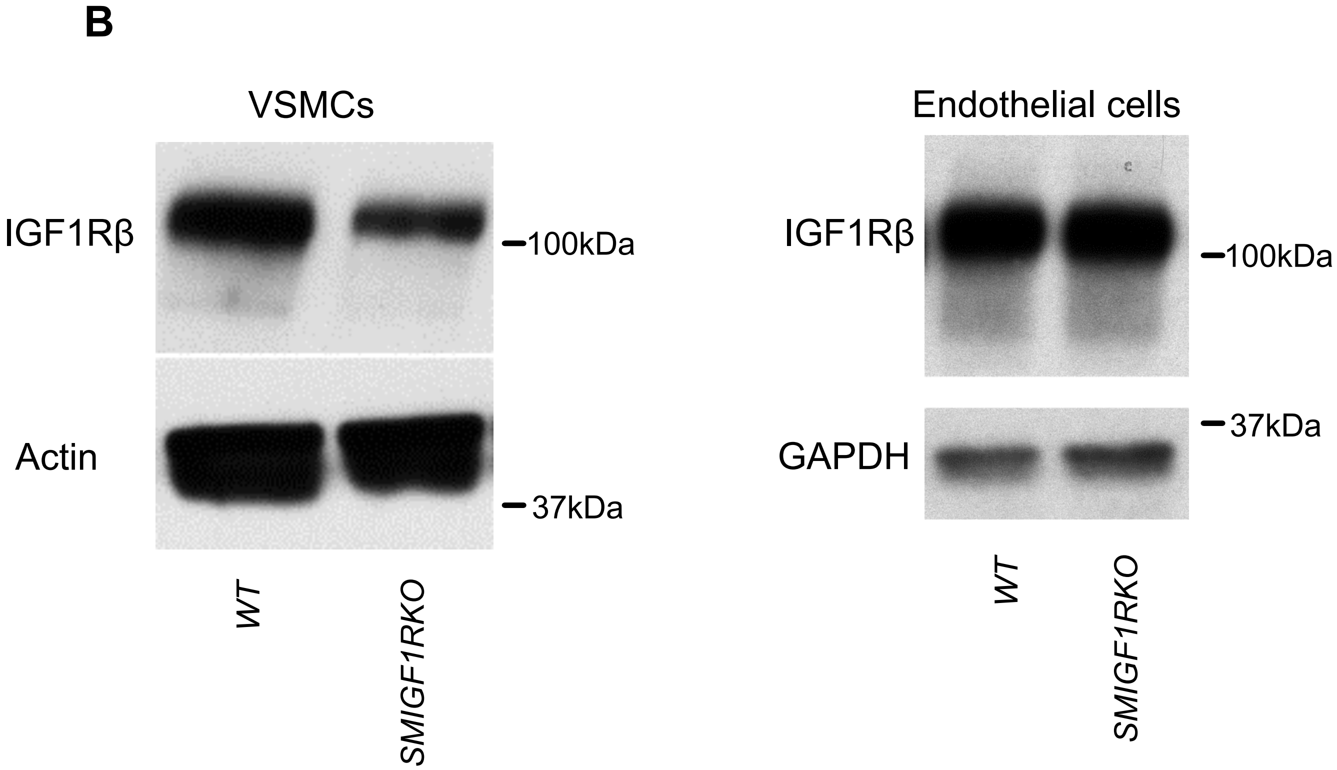

Supplementary Figure 7

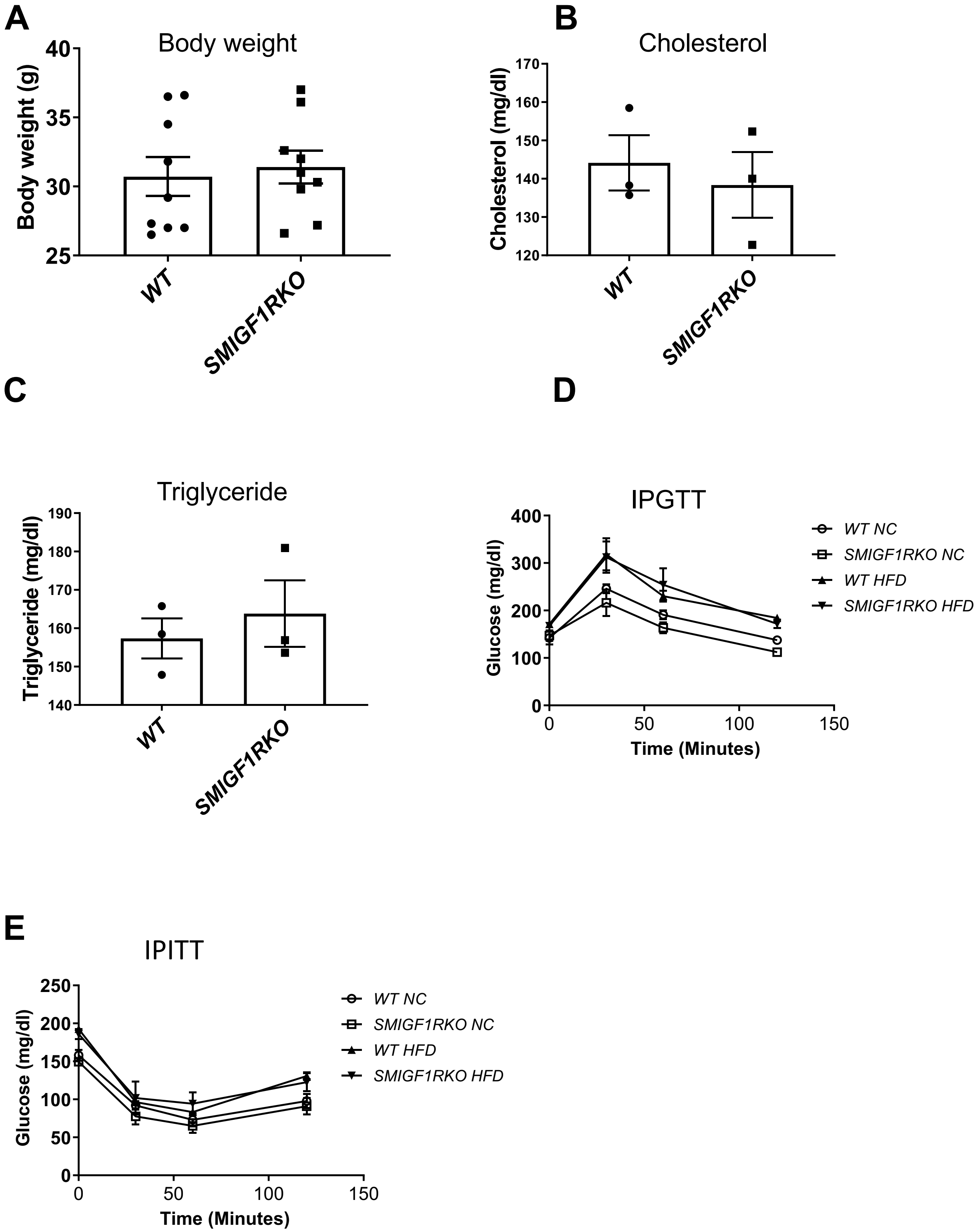

Supplementary Figure 8

A

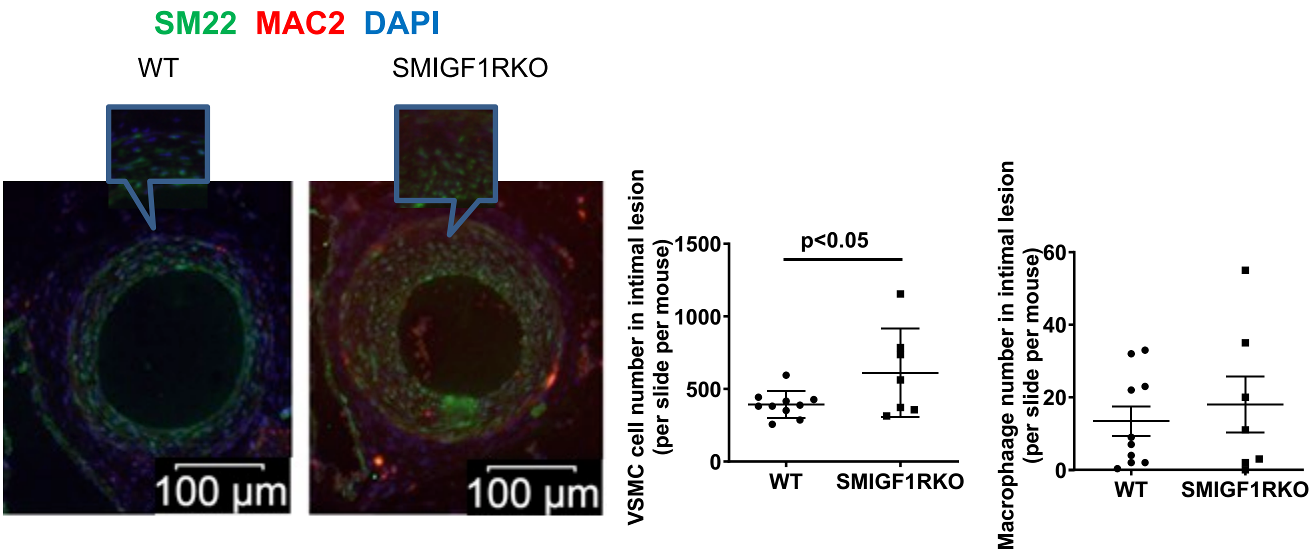

B

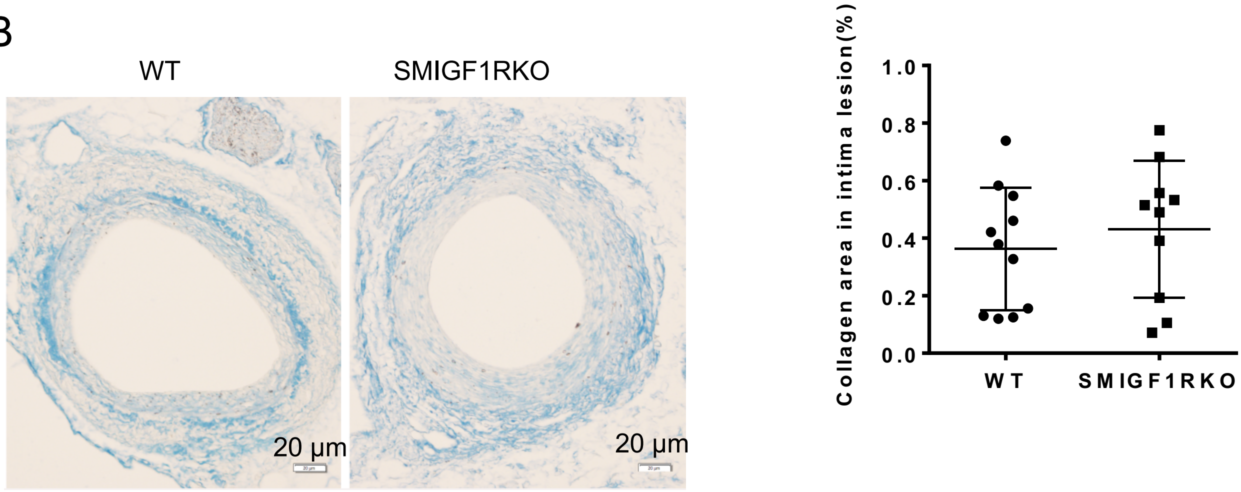

Supplementary Figure 9

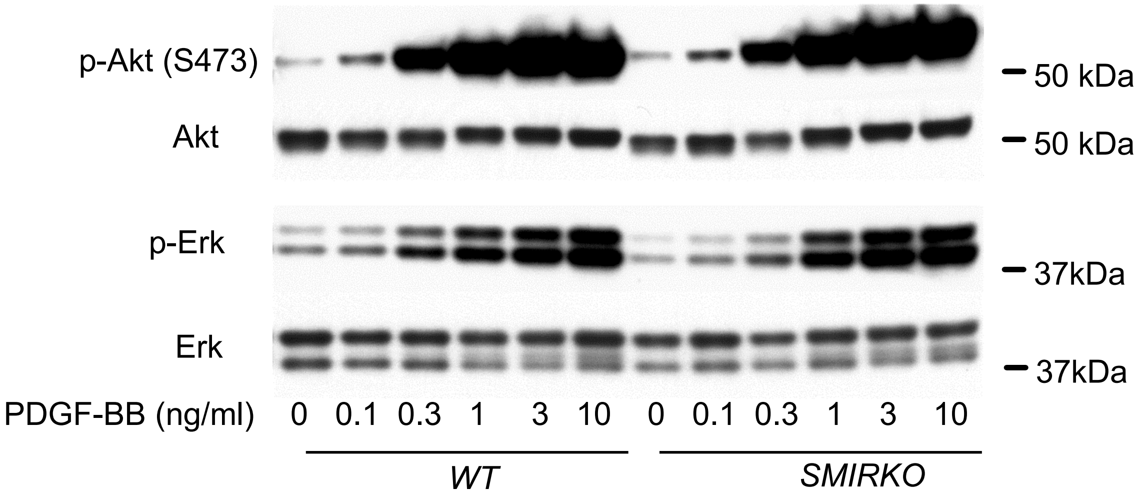

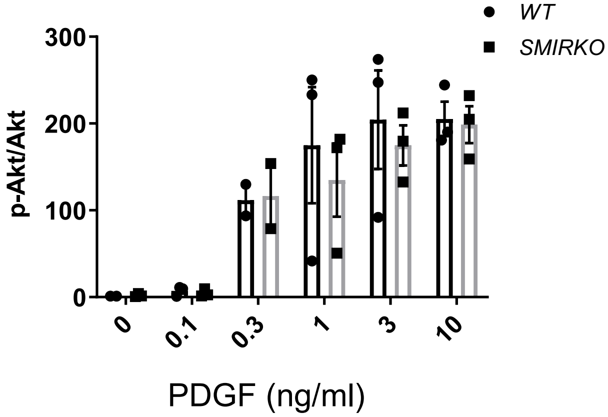

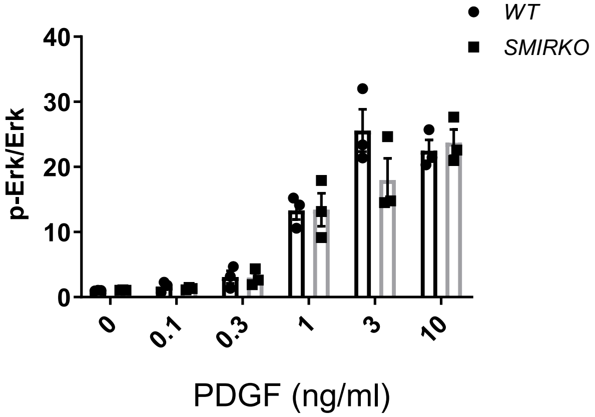

Supplementary Figure 10

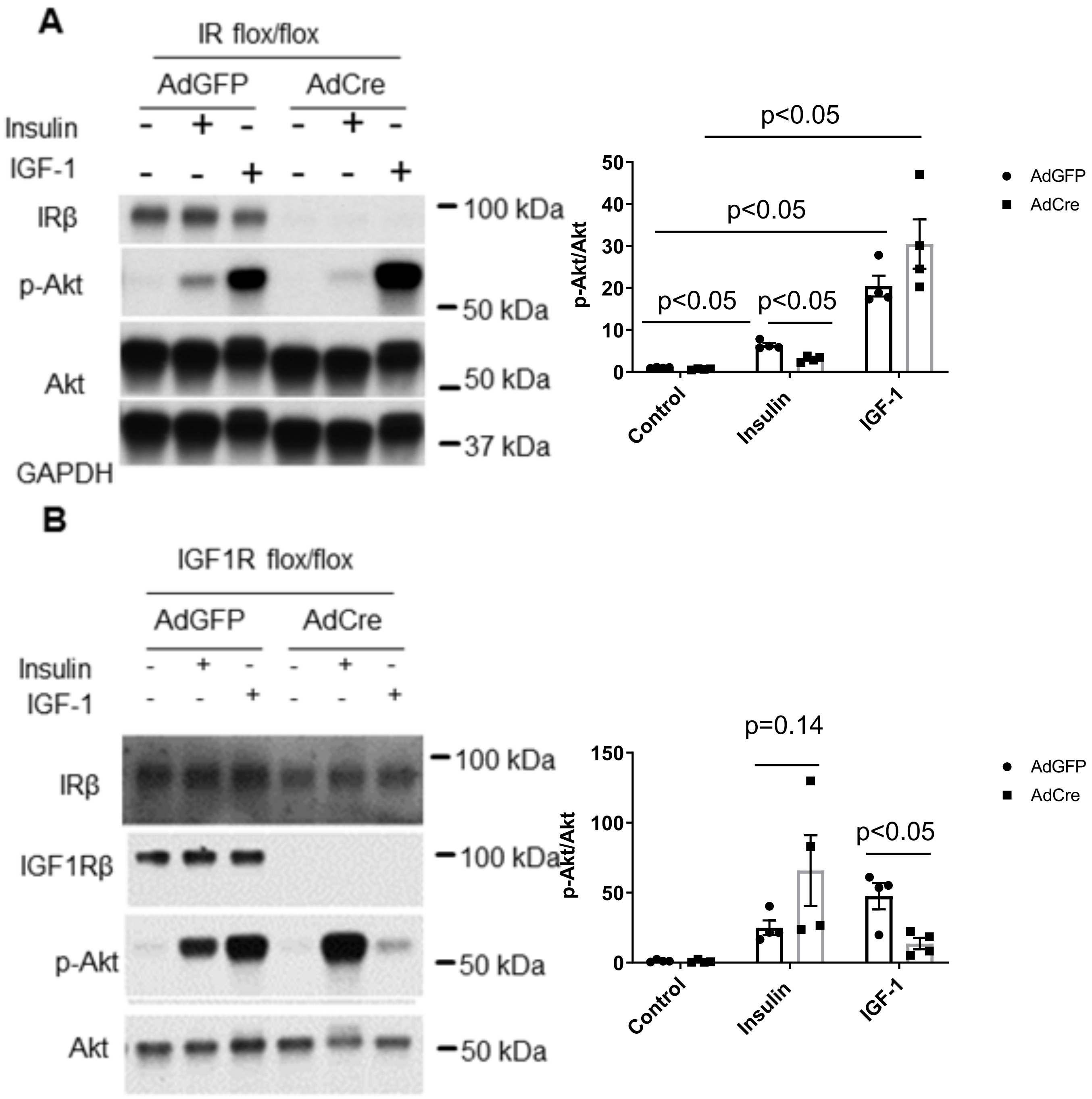

Supplementary Figure 11

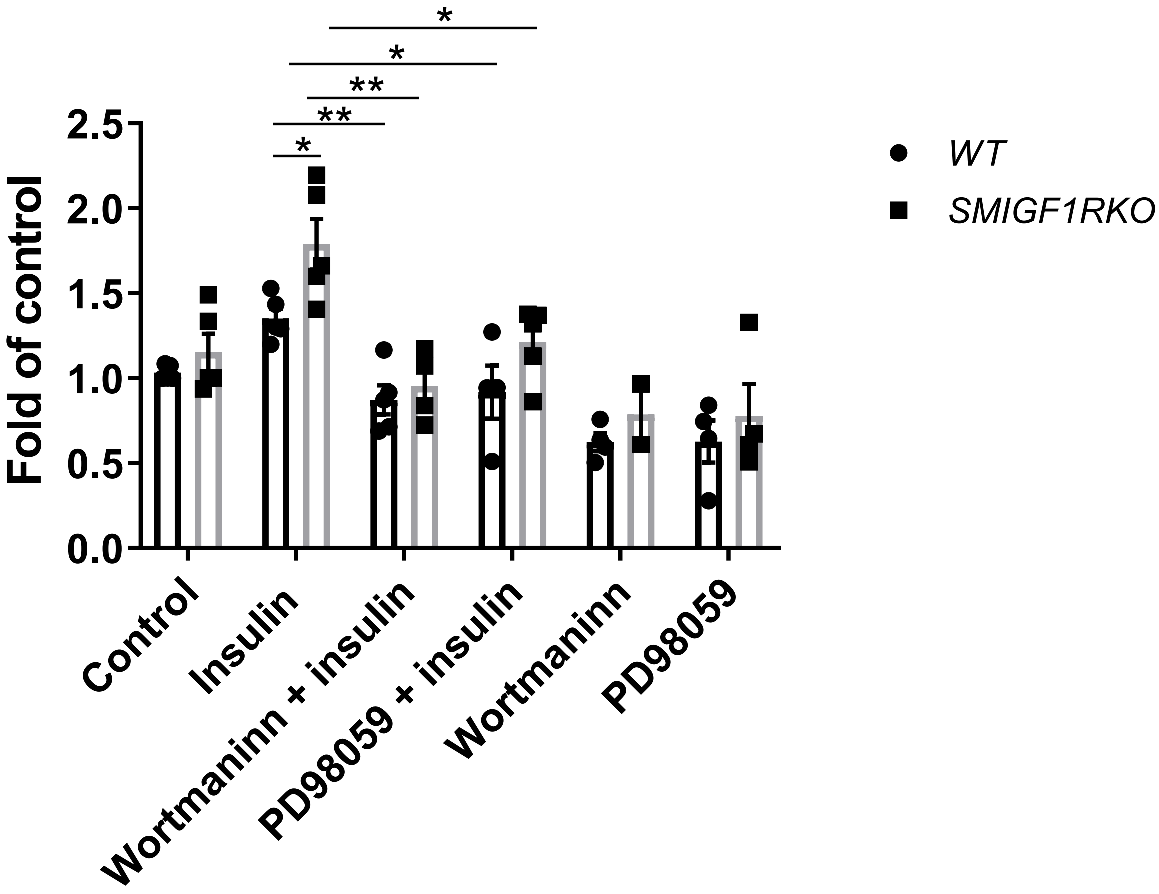

Supplementary Figure 12

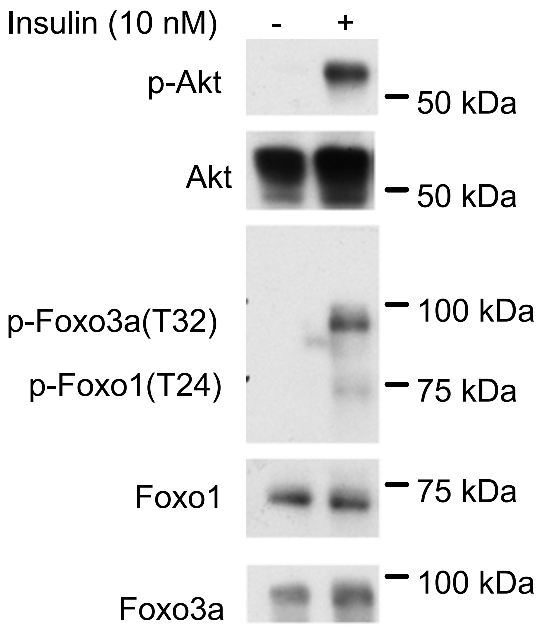

Supplementary Figure 13

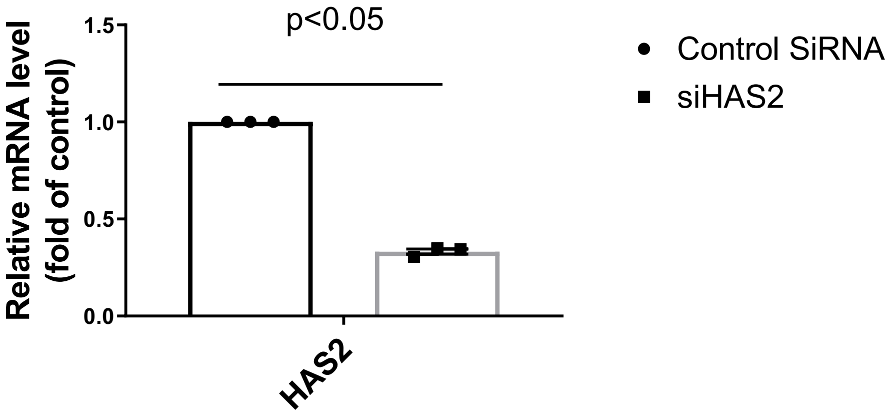

Supplementary Figure 14

A

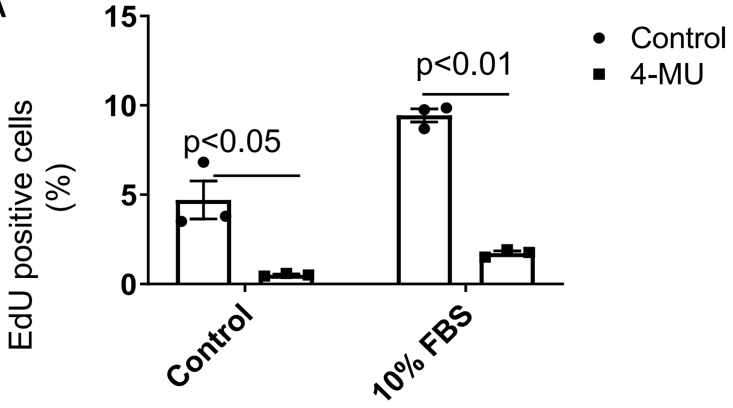

B

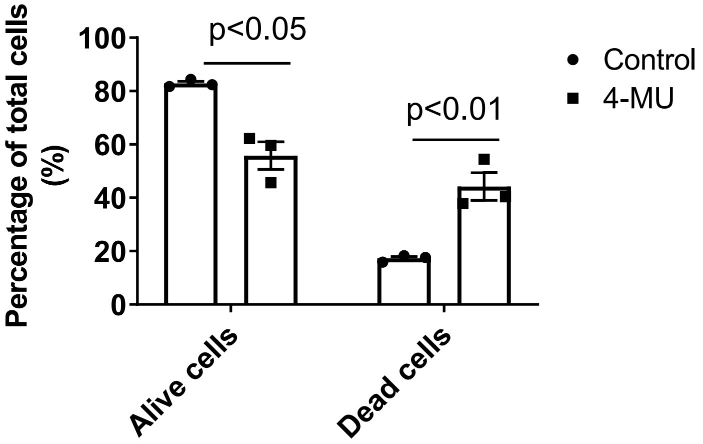

Supplementary Figure Legends

Supplementary Table 1. Genes differentially regulated by insulin in *WT* and *SMIGF1RKO* mice. VSMCs from *SMIGF1RKO* mice were transfected with AdCre to further knockdown IGF1R. Then *WT* and *SMIGF1RKO* VSMCs were treated with 100 nM insulin and RNA was extracted for RNA sequencing (n=3 per group). Source data are provided as a Source Data file.

Supplementary Figure 1. IR expression levels in different tissues and cells of *WT* and *SMIRKO* mice were determined by western blotting. Source data are provided as a Source Data file.

Supplementary Figure 2. Insulin stimulated Akt phosphorylation in *WT* and *SMIRKO* mice in vivo. The fasted mice were administrated with insulin through intravenous injection at 10 $IUKg^{-1}$ body weight. The tissues were collected at 5 minutes after insulin injection. Akt phosphorylation were determined by western blotting (n=4 per group). The data are mean ± SEM. One-way ANOVA with a post hoc test. Source data are provided as a Source Data file.

Supplementary Figure 3. Metabolic parameters of *WT* and *SMIRKO* mice on HFD. A. Body weight (*WT* n=6, *SMIRKO* n=7). B. Blood pressure (n=6 per group). C. Plasma insulin level (*WT* n=9, *SMIRKO* n=8). D. Plasma triglyceride and cholesterol (n=8 per group). E. IPGTT. F. IPITT. The data are mean ± SEM. Two tailed t test. Source data are provided as a Source Data file.

Supplementary Figure 4. Intimal hyperplasia of femoral artery induced by wire injury in HFD fed *WT* and *SMIRKO* mice. A-D. Intimal hyperplasia of femoral artery was determined by elastin staining at four weeks after wire injury. A indicated representative images. Summarized intimal area B, intima/media (I/M) ratio C and media area D were showed (*WT* n = 10, *SMIRKO* n = 11). E-F VSMC proliferation in wire-injured femoral artery. The femoral arteries of mice were subjected to wire injury and then the mice were infused with Brdu with osmotic minipump for one week. VSMCs proliferation was determined by SM22α and Brdu double staining. E indicated representative images and F showed summarized data (*WT* n = 9, *SMIRKO* n = 10). G. *Cyclin A2* gene expression in wire-injured femoral artery. *Cyclin A2* gene expression in femoral artery at 9 day after wire injury was used as a surrogate marker of cell proliferation and was determined by qPCR (*WT* sham n=6, *WT* injury n=7, *SMIRKO* sham n=4, *SMIRKO* injury n=8).H. Insulin and IGF-1 induced VSMCs proliferation. Starved VSMCs were stimulated with insulin (10 nM) or IGF-1 (10 nM) for 24 hours and cell proliferation was determined by Edu incorporation and measured by flow cytometry (*WT* control n=9, *WT* insulin n=9, *WT* IGF-1 n=6, *SMIRKO* control n=9, *SMIRKO* insulin n=9, *SMIRKO* IGF-1 n=6). The data are mean ± SEM. Two tailed t test or two-way ANOVA with a post hoc test. Source data are provided as a Source Data file.

Supplementary Figure 5. IRβ expression in different tissues. Source data are provided as a Source Data file.

Supplementary Figure 6: IGF1R expression levels in different tissues and cells of WT and SMIGF1RKO mice were determined by western blotting. Source data are provided as a Source Data file.

Supplementary Figure 7. Metabolic parameters of *WT* and *SMIGF1RKO* mice. A. Body weight (n=9 per group). B. Plasma cholesterol (n=3 per group). C. Plasma triglyceride (n=3 per group). D. IPGTT. Two tailed t test. Source data are provided as a Source Data file.

Supplementary Figure 8. A. VSMCs and macrophage staining. The femoral arteries were subjected to wire injury and VSMCs and macrophage staining were performed at 4 weeks after injury. B. Collagen

staining. The femoral arteries were subjected to wire injury and collagen staining was performed at 4 weeks after injury. Two tailed t test. Source data are provided as a Source Data file.

Supplementary Figure 9. PDGF signaling in VSMCs from *WT* or *SMIRKO* mice. The cells were treated with different dose of PDGF for 10 minutes. Akt and Erk phosphorylation were determined by western blotting (n=3 per group). The data are mean ± SEM. Two-way ANOVA with a post hoc test. Source data are provided as a Source Data file.

Supplementary figure 10. Insulin and IGF-1 actions on IR or IGF1R knockdown VSMCs. A. IR flox/flox VSMCs were infected with AdCre to knockdown IR. Akt phosphorylation after 10 nM insulin or IGF-1 stimulation were determined by western blotting (n=4 per group). B. . IGF1R flox/flox VSMCs were infected with AdCre to knockdown IGF1R. Akt phosphorylation after 100 nM insulin or IGF-1 stimulation were determined by western blotting (n=4 per group). The data are mean ± SEM. Two-way ANOVA with a post hoc test. Source data are provided as a Source Data file.

Supplementary figure 11. Insulin induced Has2 expression through both PI3K and MAPK pathway. VSMCs were pretreated with wortmannin or PD98059 for 30 minutes and then stimulated with 10 nM insulin for 4 hours. Two-way ANOVA with a post hoc test. Source data are provided as a Source Data file.

Supplementary Figure 12. Insulin induced FoxO phosphorylation in VSMCs. Starved VSMCs were stimulated with 10 nM insulin for 15 minutes. Akt and FoxO phosphorylation were determined by Western blotting . Source data are provided as a Source Data file.

Supplementary Figure 13. Knockdown of *Has2* gene expression. VSMCs were transfected with siRNA targeted to Has2 (n=3 for each group). The data are mean ± SEM. Two tailed t test. Source data are provided as a Source Data file.

Supplementary Figure 14. 4-MU inhibited cellular proliferation and induced cell apoptosis. A. VSMCs were treated with 1mM 4-MU for 4 hours and then treated with 10% FBS for 24 hours. Cellular proliferation was determined by EdU incorporation. (n=3 for each group, The data are mean ± SEM.). B. VSMCs were treated with 1mM 4-MU for 24 hours and apoptosis was determined by Annexin V and PI double staining. (n=3 for each group, The data are mean ± SEM.). Two tailed t test or two-way ANOVA with a post hoc test. Source data are provided as a Source Data file.
