# Supplementary material for: Homozygous receptors for insulin and not IGF-1 accelerate intimal hyperplasia in insulin resistance and diabetes
Source: Nat Commun. 2019 Sep 27;10:4427. doi: 10.1038/s41467-019-12368-2 (PMC6765023; doi:10.1038/s41467-019-12368-2)
Supplement: Supplementary file 2 — Description of Additional Supplementary Files [file 41467_2019_12368_MOESM2_ESM.docx]

Supplementary dataset

**Read-counts of RNA-sequencing experiments.** RNA-sequencing experiments were performed with aortic VSMCs from WT or SMIGF1RKO mice stimulated with 100 nM insulin for 4 hours (n=3 per group). Sequencing reads from FASTQ files were aligned to the reference genome using the RNA-specific STAR aligner to generate sequence alignment (BAM) files. The featureCounts tool was then used taking in the BAM files to count sequencing reads mapping to the reference genome at the gene level. The read counts were subsequently normalized between samples using DESeq.
